# Knowledge Gaps in End-of-Life Care and Planning Options Among Older Adults in Switzerland

**DOI:** 10.3389/ijph.2022.1604676

**Published:** 2022-08-25

**Authors:** Sarah Vilpert, Gian Domenico Borasio, Jürgen Maurer

**Affiliations:** ^1^ Faculty of Business and Economics (HEC), University of Lausanne, Lausanne, Switzerland; ^2^ Swiss Centre of Expertise in the Social Sciences, Lausanne, Switzerland; ^3^ Palliative and Supportive Care Service, Lausanne University Hospital (CHUV) and University of Lausanne, Lausanne, Switzerland

**Keywords:** older adults, knowledge, palliative care, advance directives, assisted suicide

## Abstract

**Objectives:** Good knowledge about end-of-life (EOL) care options helps in discussing and planning important aspects of the end of life in advance and contributes to improved well-being among dying patients and their families.

**Methods:** Our study explores knowledge levels of EOL care and planning options and its sociodemographic and regional patterning using nationally representative data from respondents aged 55+ of wave 6 of the Survey of Health, Ageing and Retirement in Europe in Switzerland (*n* = 2,199).

**Results:** Respondents answered correctly on average to just under four out of eight questions regarding EOL care options. Women, individuals with higher education levels, and those living with a partner showed a higher EOL knowledge score, whereas the score is lower among older adults (75+) and individuals living in French- and Italian-speaking Switzerland.

**Conclusion:** In view of the significant EOL knowledge gaps among older adults in Switzerland, further education efforts on EOL care options are needed, with particular attention to the population groups most affected.

## Introduction

At the end of life, patients and/or their families are often called upon to make important medical decisions such as whether to forgo a potentially life-prolonging but perhaps otherwise challenging treatment [1]. Discussions around medical end-of-life (EOL) decisions often take place very late in the development of the disease [2], as physicians often do not feel the necessity to start the discussion with patients feeling well, even when terminally ill [3]. However, when discussions about medical EOL decisions occur earlier, they are more commonly associated with less aggressive EOL care [4, 5] and greater patient quality of life [5].

Initiating a discussion about EOL care with one’s physician or completing advance directives requires awareness of EOL care issues and options. Increased knowledge about available EOL care and planning options helps in discussing and planning self-rated important aspects of the end of life, in anticipation of a disease, with key stakeholders such as general practitioners and close relatives [6]. In addition, good knowledge of EOL care and options potentially enables individuals to have better control over their end of life [7] and experience higher quality of end of life care [8]. Finally, adequate knowledge on EOL medical treatments allows for informed decision-making and avoids misconceptions that lead to choices that do not correspond to individual values [9].

To date, little is known about the level of information about available EOL care options in the general population. Indeed, only few existing studies evaluate individuals’ knowledge of EOL options in health care [10–13] using test-based knowledge assessments. These studies typically surveyed patients or the general public in North America and assessed their knowledge related to patients’ rights to refuse or withdraw treatment, the legality of euthanasia and physician-assisted suicide, the definition of a living will and durable power of attorney. They generally showed relatively low levels of knowledge of EOL care issues in their study populations.

Moreover, potential gaps in knowledge about EOL care options among certain population groups may generate inequalities at the end of life. Thus, there is an interest in identifying the characteristics of individuals who are less informed about available EOL care options. Increasing the level of EOL care knowledge in these especially vulnerable groups would ensure that everybody benefits from equal access to adequate EOL care.

Our study aims to measure EOL care knowledge among adults aged 55+ in Switzerland with regard to the use of advance directives, palliative care, and euthanasia/assisted suicide, using a test-based assessment. In addition, we explore social and regional patterning of knowledge about EOL care and planning options to identify a possible EOL care knowledge gap in specific population groups.

## Methods

### Data

The data used in this study come from a paper-and-pencil self-completion questionnaire about EOL preferences, knowledge, attitudes and behaviors [14] that was developed by the authors. The EOL questionnaire was administered as part of the 2015 data collection round (wave 6) of the Swiss component study of the Survey of Health, Ageing and Retirement in Europe (SHARE) [15]. SHARE is a longitudinal, interdisciplinary and cross-national data infrastructure that comprises individual-level information on the health, socio-economic status, social and family networks of older persons from 27 European countries and Israel. The Swiss SHARE study was approved by the ethics committee of the canton of Vaud in March 2014 (approval number 66/14). The Swiss SHARE sample is designed to be nationally representative of community-dwelling individuals aged 50 and older and their partners.

In SHARE wave 6, 2,806 respondents participated in Switzerland and 94% of those also completed our EOL questionnaire, which resulted in a raw sample of 2,630 respondents. As the Swiss SHARE sample was last refreshed in 2011, only adults aged 55 and over were retained for our analyses, since respondents aged 50–54 in 2015 could only enter SHARE as partners and are therefore not representative of the general population aged 50–54. Retaining only observations of individuals aged 55 and over and with no missing data on all variables used in our analysis results in a final analytical study sample of 2,199 respondents.

### Outcomes

We asked respondents to assess eight statements related to EOL options as true or false. A “don’t know” response option was also offered to respondents to minimize the impact of potential guessing or item non-response. The first four statements concerned aspects around the use of advance directives in Switzerland, which were followed by two statements measuring respondents’ knowledge regarding palliative care and two statements assessing individuals’ knowledge concerning euthanasia and assisted suicide in Switzerland. Complete English translations of all eight statements along with their correct answer categories are presented in Table 2. All eight statements were individually transformed into dichotomic variables: correct answers were coded 1, and wrong or “don’t know” answers were coded 0. In addition to analyzing each knowledge item individually, we also generate a summary score for respondents’ overall knowledge around EOL issues by counting the number of correct responses of each individual, which yields a knowledge score that ranges from zero (“no correct response”) to eight (“all responses correct”).

Our independent variables include: sex; age; education levels; living or not with a partner in the same household; having living children; living in an urban or rural areas; and living in the German-, French- or Italian-speaking parts of Switzerland, as measured by the language used during the interview. Age is considered in three groups: 55–64; 65–74; 75+. Education levels are grouped in three categories based on the ISCED1997 classification [16]: elementary and lower secondary education (ISCED levels 0–2) indicate “low education”, while upper- and post-secondary education (ISCED levels 3–4) and tertiary education (ISCED levels 5–6) indicate “medium education” and “high education”, respectively. The type of living area is measured based on self-reports where respondents chose between: big city; suburb or outskirts of a big city; large town; small town; rural area, village. The first four categories have been grouped as “urban area” in our study and the last category forms the “rural area.”

### Statistical Analyses

We used weighted mean and proportion estimation to assess overall and item-specific knowledge around the different EOL care options in our target population. The employed survey weights correspond to the calibrated cross-sectional individual weights provided in SHARE [17]. Associations of knowledge score of EOL care options with respondents’ sociodemographic and regional characteristics were assessed using ordinary least squares regression. Logistic regression models were used to calculate associations of each of the eight statements with respondents’ sociodemographic and regional characteristics. To facilitate the interpretation of the estimates from our non-linear logit models, we expressed our estimates in terms of average partial effects (APEs). APEs report how the average conditional outcome probability P(Y = 1 | X) changes when a given explanatory variable changes from 0 to 1, holding all other independent variables at their observed values [18]. Estimated standard errors account for clustering at the household level to correct for potential unobserved dependencies between two observations coming from the same household. All statistical analyses were conducted using Stata SE v. 15.0 (StataCorp, College Station).

## Results

Table 1 presents key characteristics of our final analytical sample. Reflecting the structure of the SHARE’s target population, about half of the respondents are female (50.1%) and aged 55–64 years (49.3%). The large majority of respondents have a medium education level (69.6%), live with their partner (70.9%), have living children (82.2%), and live in German-speaking Switzerland (73.7%).

**TABLE 1:** Main characteristics of the analytical sample, adults aged 55+ in Survey of Health, Ageing and Retirement in Europe (SHARE), Switzerland, 2015 (*n* = 2,199).

	Weighted proportion %	(95%-CI)
Social characteristics		
Women	50.1	(48.0, 52.1)
Age groups		
55–64	49.3	(46.7, 51.9)
65–74	27.9	(25.9, 30.0)
75+	22.8	(20.8, 24.7)
Education level		
Low education	13.0	(11.5, 14.5)
Medium education	69.6	(67.4, 71.8)
High education	17.4	(15.5, 19.3)
Partner living in household	70.9	(68.5, 73.2)
Having children	82.2	(80.2, 84.2)
Regional characteristics		
Urban area	47.7	(45.1, 50.3)
Linguistic region		
German-speaking	73.7	(71.4, 76.1)
French-speaking	23.6	(21.3, 25.8)
Italian-speaking	2.7	(1.9, 3.5)

**FIGURE 1:**
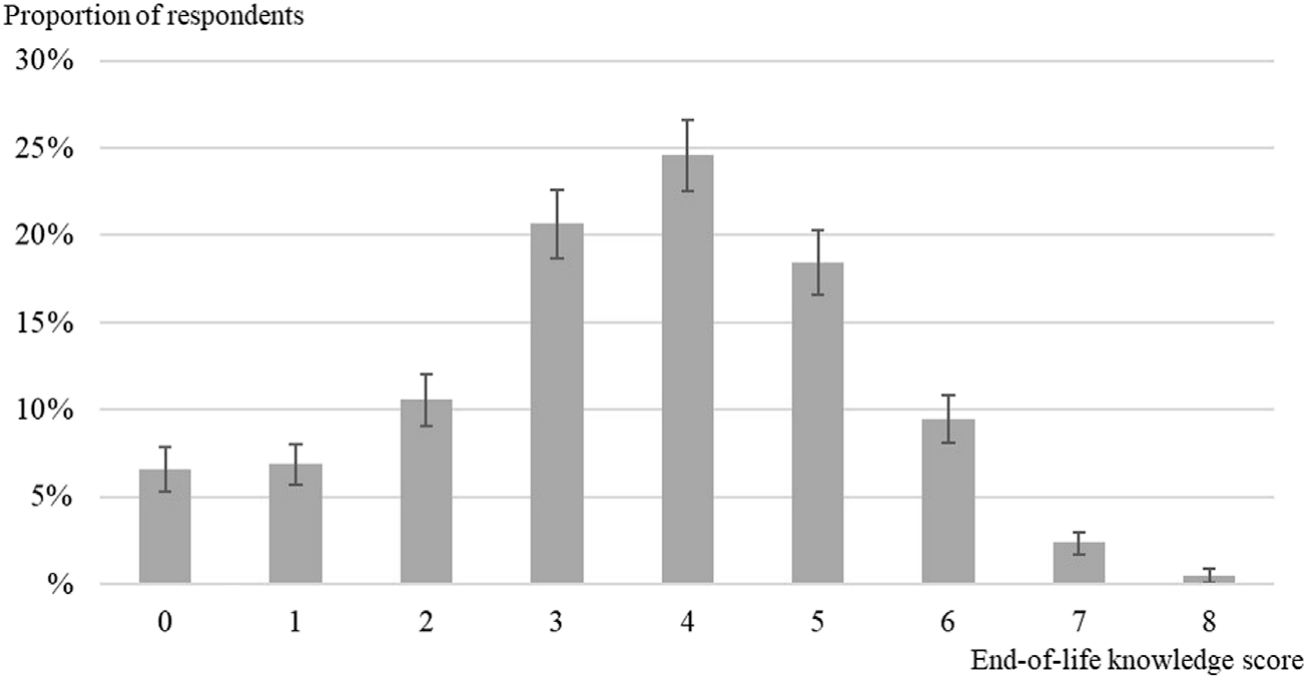
Distribution of the knowledge score of end-of-life care and planning options among adults aged 55+ in Survey of Health, Ageing and Retirement in Europe (SHARE), Switzerland, 2015 (*n* = 2,199).

The estimated mean of the knowledge score of EOL care and planning options is 3.6, with a standard deviation of 1.7. Figure 1 shows the distribution of the knowledge score of EOL options. Whereas 7% of respondents answered either incorrectly or “don’t know” to all of the eight statements (score = 0), 0.5% of respondents (10 cases) assessed correctly the eight statements (score = 8). About every second respondent appraised properly at least half of the statements (score = 4 and more).

As shown in Table 2, looking at the different knowledge items individually revealed considerable differences in respondents’ knowledge of EOL options. Specifically, respondents generally appear to be better informed about practical aspects regarding advance directives than about issues or concepts around palliative care and euthanasia/assisted suicide.

**TABLE 2:** Statements about end-of-life care and planning options, adults aged 55+ in Survey of Health, Ageing and Retirement in Europe (SHARE), Switzerland, 2015 (*n* = 2,199).

	Weighted Proportion %		(95%-CI)
Knowledge regarding advance directives			
In Switzerland, people can plan how their possessions and medical situation should be handled if they become incapable of making decisions due to disease or accident. Are the following statements about current law in Switzerland true or false?			
In Switzerland, …			
… it is legally possible to name somebody as one’s healthcare proxy	**True**	67.7	(65.4, 70.0)
	False	7.1	(5.9, 8.4)
	Don’t know	25.1	(23.0, 27.2)
… the closest relative is in charge of medical decisions for an incapacitated person if this person did not name anybody in advance	**True**	53.6	(51.3, 56.0)
	False	19.0	(17.1, 20.9)
	Don’t know	27.4	(25.3, 29.5)
… a physician can continue a treatment that the patient has refused in writing (advance directive) if the physician thinks that the treatment is necessary to prolong the patient’s life	True	10.6	(9.1, 12.1)
	**False**	65.0	(62.7, 67.3)
	Don’t know	24.4	(22.3, 26.4)
… it is possible to indicate on one’s health insurances card that one has completed a document about one’s wishes and refusals for medical treatment (advance directives)	**True**	47.5	(45.1, 50.0)
	False	12.8	(11.2, 14.4)
	Don’t know	39.7	(37.4, 42.1)
Knowledge regarding euthanasia, assisted suicide and palliative care			
To your knowledge, are the following statements about medical care at the end of life true or false?			
Patients with advanced dementia can make use of assisted suicide as long as they have clearly expressed this wish in their advance directives	True	44.3	(41.9, 46.7)
	**False**	22.1	(20.2, 24.1)
	Don’t know	33.5	(31.3, 35.8)
In Switzerland, doctors are not allowed to directly inject a lethal substance to a patient in order to end his life, even if he asks them to do so	**True**	65.6	(63.3, 67.9)
	False	15.3	(13.6, 17.0)
	Don’t know	19.1	(17.2, 21.0)
Palliative care means stopping all medical treatment and giving morphine to ensure a peaceful death	True	67.9	(65.6, 70.2)
	**False**	10.5	(9.1, 11.9)
	Don’t know	21.6	(19.5, 23.7)
Palliative care should start early in the disease course and can prolong life significantly	**True**	25.7	(23.6, 27.7)
	False	34.1	(31.8, 36.4)
	Don’t know	40.3	(37.9, 42.6)

Response categories in bold are the correct responses.

A majority of respondents correctly assessed each of the advance directives-related knowledge items. While “don’t know” is the second most frequent response for all advance directives-related knowledge items (ranging from 24.4% to 39.7%), there are also between 7.1% and 19.0% of respondents who assessed the corresponding advance directives-related knowledge items outright incorrectly. With regard to euthanasia and assisted suicide, a majority of respondents (65.6%) correctly identified euthanasia as illegal in Switzerland. However, only 22.1% of respondents knew that persons with advanced dementia cannot make use of assisted suicide, even if they had asked for it in their advance directive. Regarding the knowledge items related to palliative care, a minority of respondents (10.5%) correctly identified the statement “palliative care means stopping all medical treatment and giving morphine to ensure a peaceful death” as wrong. Furthermore, only 25.7% of respondents knew that “palliative care should start early in the disease course and can prolong life significantly”. Finally, a high range of respondents (19.1%–40.3%) indicated that they did not know the correct answer to statements related to euthanasia, assisted suicide or palliative care.

Table 3 shows the results of multivariable linear regression models for the knowledge score of EOL care and planning options as well as APEs from multivariable logistic regression models for each of the eight statements about EOL options using the aforementioned respondents’ sociodemographic and regional characteristics as explanatory variables. Holding other characteristics constant, the knowledge score is on average 0.4 points higher among women compared to men (*p* < 0.001). Individuals with medium and high education levels have 0.5 points (*p* < 0.001) and 0.8 points (*p* < 0.001) higher knowledge scores on average when compared to individuals with a low education level. Similarly, respondents living with a partner score on average 0.3 points higher than respondents who live alone (*p* < 0.001). Conversely, adults aged 75+ show a lower knowledge score (−0.3 points, *p* < 0.01) compared to adults aged 55–64. People living in the French- (−0.9 points, *p* < 0.001) and Italian-speaking (−0.8 points, *p* < 0.001) parts of Switzerland also displayed lower knowledge scores compared to individuals from German-speaking Switzerland.

**TABLE 3:** Ordinary least square regression of knowledge score and average partial effects (APEs) based on logistic regressions of knowledge of end-of-life care and planning options on sociodemographic and regional characteristics, in adults aged 55+ in Survey of Health, Ageing and Retirement in Europe (SHARE), Switzerland, 2015 (*n* = 2,199).

		Advance directives				Assisted suicide and euthanasia		Palliative care	
	Knowledge score	AD1	AD2	AD3	AD4	AS1	AS2	PC1	PC2
	b/(ci95)	APE/(ci95)	APE/(ci95)	APE/(ci95)	APE/(ci95)	APE/(ci95)	APE/(ci95)	APE/(ci95)	APE/(ci95)
Social characteristics									
Female	0.4*** (0.2, 0.5)	6.3** (2.5, 10.1)	7.8*** (3.6, 12.0)	7.0*** (3.0, 11.0)	0.6 (−3.2, 4.5)	4.2* (0.8, 7.5)	8.3*** (4.3, 12.3)	0.3 (−2.2, 2.9)	1.2 (−2.5, 4.9)
Age groups									
55–64 (ref.)	—	—	—	—	—	—	—	—	—
65–74	−0.1 (−0.3, 0.1)	−2.5 (−7.0, 2.0)	−5.6* (−10.5, −0.8)	−1.5 (−5.9, 3.0)	−2.4 (−7.2, 2.4)	−1.5 (−5.6, 2.6)	−4.6 (−9.3, 0.1)	1.7 (−1.4, 4.8)	5.7** (1.4, 10.0)
75+	−0.3** (−0.5, −0.1)	−6.4* (−11.7, −1.1)	−3.1 (−8.6, 2.5)	−5.9* (−11.1, −0.7)	1.6 (−3.8, 7.0)	−6.7** (−11.3, −2.2)	−11.7*** (−17.1, −6.4)	−3.7* (−7.0, −0.4)	8.1** (3.1, 13.2)
Education level									
Low education (ref.)	—	—	—	—	—	—	—	—	—
Medium education	0.5*** (0.2, 0.7)	7.9* (1.6, 14.2)	8.5* (2.0, 15.0)	6.2 (−0.2, 12.5)	2.8 (−3.4, 8.9)	7.0** (2.4, 11.7)	10.5** (4.1, 16.8)	0.6 (−3.1, 4.3)	2.8 (−2.6, 8.3)
High education	0.8*** (0.5, 1.1)	13.2*** (5.8, 20.6)	11.4** (3.5, 19.3)	12.7*** (5.2, 20.3)	1.8 (−5.8, 9.3)	14.4*** (8.1, 20.7)	12.7** (5.0, 20.4)	8.6*** (3.5, 13.8)	3.1 (−3.9, 10.0)
Partner living in household	0.3*** (0.1, 0.5)	5.4* (0.4, 10.5)	10.5*** (5.2, 15.7)	6.3* (1.2, 11.4)	4.1 (−1.0, 9.3)	3.0 (−1.2, 7.3)	2.5 (−2.5, 7.5)	1.9 (−1.4, 5.2)	−0.2 (−4.9, 4.4)
Having children	0.1 (−0.1, 0.3)	2.6 (−2.8, 8.1)	4.7 (−1.1, 10.5)	0.9 (−4.8, 6.5)	0.8 (−4.8, 6.4)	−2.4 (−7.4, 2.6)	5.5 (−0.3, 11.2)	−1.0 (−4.8, 2.8)	−3.3 (−8.6, 2.0)
Regional characteristics									
Urban area	0.1 (−0.0, 0.3)	0.3 (−3.8, 4.4)	0.3 (−4.0, 4.6)	3.4 (−0.6, 7.5)	2.4 (−1.8, 6.7)	2.0 (−1.6, 5.7)	2.1 (−2.0, 6.2)	1.4 (−1.4, 4.1)	0.8 (−3.1, 4.7)
Linguistic region									
German-speaking (ref.)	—	—	—	—	—	—	—	—	—
French-speaking	−0.9*** (−1.0, −0.7)	−19.3*** (−24.5, −14.0)	−7.2** (−12.4, −1.9)	−19.9*** (−24.9, −14.9)	−38.6*** (−42.9, −34.3)	−6.4** (−10.4, −2.4)	−4.0 (−8.9, 0.9)	7.6*** (3.9, 11.4)	2.2 (−2.5, 6.9)
Italian-speaking	−0.8*** (−1.2, −0.4)	−19.6** (−31.6, −7.6)	5.4 (−6.7, 17.5)	−7.5 (−20.7, 5.8)	−36.3*** (−47.2, −25.3)	−3.9 (−13.2, 5.4)	−7.0 (−19.2, 5.2)	−1.7 (−8.2, 4.8)	−6.7 (−15.9, 2.6)
*n*	2199	2199	2199	2199	2199	2199	2199	2199	2199

Average partial effects and their 95%-CI based on logistic regression are multiplied by 100.

Asterisks indicate levels of significance: ***<0.1%, **<1%, *<5%.

Knowledge score: a correct response gives one point, whereas a wrong or “don’t know” response gives zero point. The knowledge score is the sum of the eight knowledge questions.

Interpretation of knowledge score: predicted knowledge score of EOL options is 0.4 point higher among women compared to men.

Interpretation of APEs: Being a woman increases the probability of knowing that it is allowed to legally name somebody as one’s healthcare proxy by 6.3 percentage points compared to being a man.

AD1: In Switzerland, it is allowed to legally name somebody as one’s healthcare proxy. (true).

AD2: In Switzerland, the closest relative is in charge of medical decisions for an incapacitated person if this person did not name anybody in advance. (true).

AD3: In Switzerland, a physician can continue a treatment that the patient has refused in writing (advance directive) if the physician thinks that the treatment is necessary to prolong the patient’s life. (false).

AD4: In Switzerland, it is possible to indicate on one’s health insurances card that one has completed a document about one’s wishes and refusals for medical treatment (advance directives). (true).

AS1: Patients with advanced dementia can make use of assisted suicide as long as they have clearly expressed this wish in their advance directive. (false).

AS2: In Switzerland, doctors are not allowed to directly inject a lethal substance to a patient in order to end his life, even if he asks them to do so. (true).

PC1: Palliative care means stopping all medical treatment and giving morphine to ensure a peaceful death. (false).

PC2: Palliative care should start early in the disease course and can prolong life significantly. (true).

Exploring the multivariable associations of each knowledge item individually, we find that associations between individual items of knowledge about EOL care and planning options, on one hand, and individuals’ sociodemographic and regional characteristics, on the other hand, closely follow the sociodemographic and regional patterning of knowledge score of EOL options described above. Nevertheless, a few of these associations were statistically insignificant. Exceptions concern the sociodemographic and regional patterning of knowledge regarding palliative care, which mostly displayed statistically insignificant associations with our set of explanatory variables. In addition, contrary to the negative associations observed between older age and knowledge score, older adults (65+) are more likely to correctly assess the statement “palliative care should start early in the disease course and can prolong life significantly” (APE 65–74: 5.7, *p* < 0.01; APE 75+: 8.1, *p* < 0.01) than middle-aged adults (55–64). Similarly, individuals from French-speaking Switzerland are more likely to declare the statement “palliative care means stopping all medical treatment and giving morphine to ensure a peaceful death” (APE: 7.6, *p* < 0.001) as false, whereas French-speaking Switzerland is negatively associated with knowledge score.

## Discussion

In the context of aging societies, ensuring high levels of knowledge of EOL care and planning options in the older population appears to be essential to achieve patient-centered EOL care and an as high as possible quality of life at the end of life. Our study documents major gaps in EOL care knowledge in large parts of the older Swiss population.

### Substantial Knowledge Gaps Regarding Surrogate Decision-Making in End-of-Life Care

Despite the common occurrence of surrogate decision-making at the end of life, our study shows that about one-fourth of our respondents are not aware of the option to designate a healthcare proxy in advance, and that 7.1% of respondents think that such an advanced designation of a healthcare proxy is not even allowed. In addition, more than a quarter of respondents are unaware that their closest relative would automatically become their healthcare proxy should they become incapacitated, while almost 20% of the respondents wrongly believe that this would not be the case. A Dutch study showed that a month before death, 27% of people had limited decision-making capacity and this proportion rose to 67% in the week prior to death [19]. Furthermore, a study considering non-sudden and expected deaths in German-speaking Switzerland revealed that 82.3% of deaths in 2013 were preceded by at least one of the following EOL practices: forgoing life-prolonging treatment (49.3%); intensified alleviation of pain or symptoms (29.8%); physician-assisted death (euthanasia, assisted suicide, or ending of life without the patient’s explicit request) (3.1%) [1]. In addition, in 72.2% either patient or relatives were involved in decision-making concerning these EOL practices.

These studies highlight that it is quite common that healthcare practitioners have to resort to a healthcare proxy for making EOL decisions, irrespective of whether this person was designated as a healthcare proxy in advance or not. Thus, individuals should be aware at a minimum that their next-of-kin might be involved in surrogate decision-making at their end of life. This would give them the opportunity to prepare the legally designated healthcare proxies for this eventuality through discussion of their preferences and potential refusal for medical treatments in advance in addition to the completion of advance directives. This anticipation is all the more important as the burden experienced by healthcare proxies in case of need to make important EOL decisions may be especially large if they are unaware of the patient’s values and EOL wishes [20].

### Widespread Lack of Knowledge About the Legally Binding Nature of Advance Directives

Our study reveals that about one in four respondents do not know whether a physician can overrule a patient’s refusal of a certain treatment based on an advance directive in case of the patient’s mental incapacity. Moreover, one in ten respondents wrongly believe that the physician’s decision takes precedence over the patient’s advance directives. Comparable results were found in a North American study of hospitalized patients where a significant proportion of the patients with advance directives “thought they should be overridden if physicians or family members thought it best to do so” [11]. These data highlight that the full implications of institutional arrangements and legal tools aimed to enhance patient autonomy such as advance directives are not yet fully grasped by a sizable minority of older adults in Switzerland. One potential reason for this finding may be that our respondents come from older generations that have commonly experienced medical paternalism throughout their lives [21] and may therefore continue to believe that physicians opinions do (and perhaps even should) take precedence over patients’ own wishes regarding their future treatment course. This interpretation appears to also be supported by the results of our regression model: older adults are less likely to know that physicians must respect a patient’s desire to withhold or withdraw treatment irrespective of their own beliefs regarding the right course of action. Correspondingly, a study on adult population (>18 years) living in the U.S. showed that compared to younger adults, older adults (>45 years) are more likely to “rely on doctor’s knowledge and not try to find out about their condition on their own” and to “leave decisions about their medical care up to doctors” [22].

### Major Misconceptions and Lack of Awareness About Palliative Care

Our study shows particularly widespread misconceptions and lack of knowledge in the area of palliative care. Between 75% and 90% respondents have chosen either “don’t know” or the wrong answers to our palliative care knowledge test questions. This relatively low level of knowledge about palliative care is particularly concerning as increasing education and awareness of palliative care was a specific goal of the Swiss national strategy for the promotion and development of palliative care 2010–2015 [23]. At a time when palliative care is aiming to become an established medical specialty taking care of patients well in advance of the terminal phase, misconceptions in the population about what palliative care are could undermine its usefulness for the patients and families.

### Women Are Better Informed About End-of-Life Care Options Than Men

Holding other characteristics fixed, women are generally better informed about EOL care options than men. These gender differences in knowledge about EOL care options were statistically significant in the domains related to both advance directives and assisted suicide, but not in the area of palliative care. To date, women have been more involved in caring for relatives than men [24] and may acquire some knowledge about EOL care options through their caregiving activities. In addition, women often outlive their spouses, as they are often the younger person in the couple while also having a longer life expectancy than men. In view of their higher likelihood of ending up alone in later life, older women may be more likely to anticipate and prepare the EOL period by themselves, which may include—among other things—seeking information about EOL care options. This finding is consistent with evidence from other studies that report a higher completion rates of advance directives among women [25, 26].

### Despite Being Most Immediately Concerned, Older Adults Often Have Lower Levels of Knowledge About End-of-Life Care Options Than Middle-Aged Adults

Older adults (65–74 and 75+) have less knowledge about EOL care options than their middle-aged counterparts, despite their closer proximity to death. Studies measuring health literacy in European populations generally report lower average levels of health literacy in older population groups [27, 28]. This data suggest that older individuals may find it more challenging to access, understand, appraise and apply information in the context of decision-making in healthcare, disease prevention, and health promotion. This may partly explain the relatively poor level of EOL care-related knowledge among older adults in our study.

### Individuals With Higher Education Levels Have Better Knowledge About End-of-Life Care Options

Keeping other characteristics fixed, education is positively associated with knowledge about EOL care options in the older population in Switzerland. A similar education gradient was also found for health literacy with higher health literacy scores among people with higher education levels [28, 29]. Notably, higher educational levels were associated with more competences related to accessing and understanding health information in the domains of healthcare and disease prevention [29]. These findings may explain why individuals with a higher education level have also more accurate knowledge about EOL care options. In addition, there may be a higher motivation among highly educated people to acquire knowledge about EOL issues due to a greater interest in being more actively involved in medical decision-making [22, 30], including at the end of life [31].

### Lower Knowledge of End-of-Life Care Options in French- and Italian-Speaking Switzerland

Individuals living in the French- and Italian-speaking parts of Switzerland show lower levels of knowledge regarding EOL care options compared to individuals living in German-speaking Switzerland, keeping other characteristics fixed. Similar geographical differences in medical EOL decisions and place of death across the three linguistic regions in Switzerland have been observed in several studies. These regional differences may partly translate cultural differences in the way death and dying are approached. The French- and Italian-speaking regions of Switzerland also have a higher proportion of hospital deaths [32], a lower level of medical EOL decisions among non-sudden and expected deaths, the most explicit practice being forgoing life-prolonging treatment [33] and lower rates of advance directive completion [26]. These findings may indicate a more technology-oriented approach to the EOL care and potentially lower levels of acceptance of death in these regions compared to the German-speaking part of Switzerland. These attitudes that may exist among public authorities, public health actors and healthcare professionals may constitute barriers to communication and education of the community on medical EOL issues.

### Actual Knowledge, Conscious Unawareness, and Outright Misconceptions About End-of-Life Care Options and Their Scope

In our study, one to two fifths of respondents declare that they do not know the correct answer to our statements and thus display conscious unawareness. These individuals are conscious of their lack of knowledge regarding EOL care options, whereas another significant part of the respondents had misconceptions about some EOL care options. It was found that healthcare professionals want [34] but avoid engaging in discussion about EOL care [35], and tend to address this issue only once their patients are affected by a serious or terminal illness [3]. Receiving information at that time may be: 1) too late, as some sudden and unpredictable diseases such as stroke or heart attack require decision-making prior to the adverse health event; 2) insufficient, as the range of options in EOL care may not be exhaustively discussed by some healthcare professionals because of a lack of knowledge of available EOL care options [36] or personal affinity with some of them [37]. Since healthcare professionals play a key role in providing health information, they should be systematically trained to communicate appropriately and in a timely manner about EOL options to their patients.

### Limitations

An analysis of missing responses in our data set revealed that, overall, women, older adults and individuals with low or medium education levels were somewhat less likely to answer our questions. However, the proportion of missing responses in these groups was well below 10%, except in adults aged 75+ and those with the lowest education level, where non-response rates ranged between 10% and 12%. These missing responses may be systematically related to respondents’ knowledge regarding our assessment items and could therefore result in bias in our estimations. Specifically, our results based on complete cases may slightly overestimate the level of knowledge regarding EOL care options in the older population in Switzerland and especially among older adults (75+) and individuals with a low education.

Moreover, our knowledge assessment based on statements about EOL care options was quite technical and may thus have generated some misunderstanding as well. Specifically, with respect to the item “Palliative care should start early in the disease course and can prolong life significantly,” the clarification of “life-threatening disease” could have avoided any misunderstanding of the type of disease we were considering, although this item was framed in the context of EOL medical care, which was the topic of the entire EOL questionnaire. However, difficulties in grasping the meaning of complicated statements containing medical jargon may by itself give some indication of potential challenges for individuals when dealing with EOL- and other healthcare issues in a highly technical medical context.

### Conclusion

Our study highlights significant knowledge gaps with regard to EOL care and planning options in older adults in Switzerland, especially among men, older adults (65–74; 75+), individuals with low education levels, and people living in the French- and Italian-speaking parts of Switzerland. Insufficient knowledge about or outright misconceptions of available EOL care and patients’ rights at the end of life in this population may result in poor EOL care choices for themselves and/or their loved ones, as well as a lack of engagement in decision making for the end of life, despite a general openness toward advance care planning and completion of advance directives [26].

In view of the rapid ageing of the Swiss population, and corresponding increases in the projected number of deaths, it appears important to raise the level of knowledge regarding EOL care options and advance care planning in the general population, especially among the least informed groups. Different actors should be involved in increasing the level of information regarding EOL options, such as general practitioners, health promotion services, and non-governmental organizations committed to the elderly population. In addition, interventions such as public awareness campaigns using mass media [38] as well as participatory approaches using informative material [6, 39] may be implemented. These actions would participate in ensuring more patient-centered EOL care delivery, thus leading to better concordance of the care with the patients’ wishes and lowering the psychological burden of the dying process for patients and their loved ones.
